# Involvement of 5-HT_1A/1B_ receptors in the antinociceptive effect of paracetamol in the rat formalin test

**DOI:** 10.1016/j.ynpai.2018.01.004

**Published:** 2018-02-01

**Authors:** A. Roca-Vinardell, E. Berrocoso, M. Llorca-Torralba, J.A. García-Partida, J. Gibert-Rahola, J.A. Mico

**Affiliations:** a Neuropsychopharmacology and Psychobiology Research Group, Department of Neuroscience, University of Cadiz, Cadiz, Spain; b Neuropsychopharmacology and Psychobiology Research Group, Department of Psychology, University of Cadiz, Cadiz, Spain; c Centro de Investigación Biomédica en Red de Salud Mental (CIBERSAM), Instituto de Salud Carlos III, Madrid, Spain; d Instituto de Investigación e Innovación en Ciencias Biomédicas de Cadiz, INiBICA, Hospital Universitario Puerta del Mar, Cadiz, Spain

**Keywords:** Paracetamol, Formalin test, 5-HT_1A_ receptors, 5-HT_1B_ receptors, Antinociceptive effect

## Abstract

The mechanism of analgesic action of paracetamol (acetominophen) remains still unknown. However, a relationship between serotonergic system and the effect of paracetamol has been previously demonstrated. The serotonin activity in the brainstem is primarily under the control of 5-HT_1A_ somatodendritic receptors, although some data also suggest the involvement of 5-HT_1B_ receptors. To determine whether the 5-HT_1A_ and 5-HT_1B_ receptors are involved in the antinociceptive effect of paracetamol, we evaluated the effect of paracetamol (0.125–1 g/kg i.p.) followed by different antagonists [WAY 100,635 (0.8 mg/kg s.c.) and SB 216,641 (0.8 mg/kg s.c.)] or agonists [8-OH-DPAT (0.125 mg/kg s.c.) and CP 93,129 (0.125 mg/kg s.c.)] of 5-HT_1A_ and 5-HT_1B_ receptors, respectively, in the rat model of formalin-induced pain. We demonstrated that paracetamol administration showed a dose-dependent antinociceptive effect in the formalin test. WAY 100,635 (5-HT_1A_ antagonist) induced an increase in the antinociceptive effect of paracetamol at 250 mg/kg doses. Conversely, 8-OH-DPAT (5-HT_1A_ agonist) decreased the antinociceptive effect of paracetamol at 500–1000 mg/kg doses. However, SB216641 (5-HT_1B_ antagonist) modified weakly the antinociceptive effect of paracetamol at 250 mg/kg doses and CP 93,129 (5-HT_1B_ agonist) not produce a clear effect in the antinociceptive effect of paracetamol. These results suggest that the antinociceptive effect of paracetamol can be enhanced mainly by compounds having 5-HT_1A_ antagonist properties in the formalin test and maybe by 5-HT_1B_ receptors antagonists.

## Highlights

• Paracetamol has an antinociceptive action in the formalin test.
• 5-HT_1A_ and 5-HT_1B_ receptors seem to be involved in the antinociceptive effect of paracetamol.
• 5-HT_1A/B_ antagonists could improve the antinociceptive effect of paracetamol.

## Introduction

Paracetamol (acetaminophen) has been extensively studied as analgesic for pain relief in many clinical settings but its mechanism of action still is under considerable debate. Paracetamol crosses the blood brain barrier and many reports indicate that paracetamol exerts its antinociceptive activity not only peripherally, but also within the central nervous system (CNS) (Courad et al., 2001). In addition, paracetamol also exhibits antinociceptive effects in tests that are reputed to be sensitive only to central analgesics, as hot-plate test and tail-flick test (Pinardi et al., 2003, Sandrini et al., 2007), and intracerebroventricular or intrathecal administration of paracetamol have also been shown to provide antinociception (Alloui et al., 2002, Raffa et al., 2004). Paracetamol has been shown to act as a selective COX-2 inhibitor in the CNS, where the concentration of tissue peroxides is low unlike at sites of inflammation (Hinz et al., 2008, Lee et al., 2007). Also, the analgesic effects of paracetamol are attenuated by drugs that act via inhibition of serotonergic, opioid and cannabinoid systems (Pickering et al., 2006, Toussaint et al., 2010) suggesting that a number of neurotransmitter system may be involved in the central antinociceptive mechanism of paracetamol, in particular, serotonergic pathways. In support of this, different studies have shown that action of paracetamol is significantly reduced when lesions are produced in the serotonergic pathway or by inhibiting synthesis of serotonin (5-HT) in animal models (Sandrini et al., 2003, Tiippana et al., 2013). Conversely, paracetamol treatment induces a significant increase in 5-HT levels in the brainsterm (Courade et al., 2001). Another hypothesis that has surfaced is that the analgesic action of systemically administered paracetamol could be attributed to spinal 5-HT (5-HT_3_ and 5-HT_7_) receptors mediated the enhanced neurotransmitter release in the descending serotonergic pathway, which is responsible for modulation of pain at the spinal level (Dogrul et al., 2012). However, other studies report a serotonergic facilitatory modulation onto the spinal cord through 5-HT_3_ in different pain models (Bannister et al., 2015, Sikandar et al., 2012).

We have previously shown that the antinociceptive effect of tramadol, an analgesic that, like paracetamol is able to increase serotonin levels within CNS, is potentiated or antagonized respectively by a 5-HT_1A/B_ nonspecific receptor blockade or activation (Rojas-Corrales et al., 2000). Moreover, it has been shown that the antinociceptive effect of clomipramine, 5-HT and NA re-uptake inhibitor, is also enhanced by the specific blockade of 5-HT_1A_ receptors (Ardid et al., 2001). In other study, we have shown that the selective blockade of the 5-HT_1A_ or 5-HT_1B_ potentiate the antinociceptive effect of paracetamol in the hot plate test, while this antinociceptive effect of paracetamol can antagonized by specific agonist of these autoreceptors, 5-HT_1A_ and 5-HT_1B_ (Roca-Vinardell et al., 2003). The hot plate test is one of the most commonly used tests of analgesic measure of analgesic drugs that act at the level of spine and higher centres (Vogel, 2002). As both central as well as peripheral mechanisms of paracetamol has been proposed, in the current study we employed the formalin test to assess the effect of blockade or activation of 5-HT_1A_ or 5-HT_1B_ receptors, by specific antagonist or agonist, on antinociceptive action of paracetamol in rats.

## Material and methods

### Animals

Experiments were carried out on adult male Wistar rats, 200–250 body weight, under standard laboratory conditions (22 °C, 12 h light/dark cycle, lights on at 08:00 AM, food and water *ad libitum*) (n = 8–11/group). All procedures and animal handling were in accordance with the guidelines of European Commission’s directive (2010/63/EC) and Spanish Law (RD 53/2013) regulating animal research, and all the experimental protocols were approved by the Committee for Animal Experimentation at the University of Cadiz (Spain).

### Drugs

The following drugs were used: propacetamol (provided by UPSA Laboratories Spain, Bristol-Myers-Squibb Group, Madrid, Spain), N-[2-[4-(2-Methoxyphenyl)-1-piperazinyl]ethyl]-N-2-pyridinylcyclohexanecarboxamide (WAY 100,635) (Sigma, St Louis, MO, USA), N-[3-[3-(Dimethylamino)ethoxy]-4-methoxyphenyl]-2′-methyl-4′-(5-methyl-1,2,4-oxadiazol-3-yl)-[1,1′ biphenyl]-4-carboxamide (SB 216,641) (Tocris, Bristol, U.K.), 8-Hydroxy-2-(di-*n*-propylamine) tetralin (8-OH-DPAT) (Sigma, St Louis, MO, USA) and 1,4-Dihydro-3-(1,2,3,6-tetrahydro-4-pyridinyl)-5H-pyrrol[3,2-b]pyridin-5-one (CP 93,129) (Tocris, Bristol, U.K.). Control animals received saline (NaCl 0.9%).

Propacetamol is a prodrug which is completely hydrolysed to paracetamol by plasma esterases within 7 min after intravenous injection (2 g of propacetamol are equivalent to 1 g of paracetamol (Bannwarth et al., 1992). Therefore, 2 g of propacetamol was dissolved in saline and intraperitoneally administered at equivalent dosis of paracetamol of 125, 250, 500 and 1000 mg/kg at a volume injection of 1 ml/kg body weight. The others drugs were dissolved in saline and subcutaneously administered in a volume injection of 1 ml/kg body weight. WAY 100,635 and SB 216,641 were administered at dose of 0.8 mg/kg. 8-OH-DPAT and CP 93,129 were administered at dose of 0.125 mg/kg. The doses of WAY 100,635, SB 216,641 and 8-OH-DPAT were chosen based on published data (Rojas-Corrales et al., 2005, Rojas-Corrales et al., 2000). The doses of CP 93,129 were chosen on the basis of previous studies performed in our laboratory (data not published).

### Formalin test

The formalin test was performed as described Dubbuison and Dennis (Dubuisson and Dennis, 1977). Before testing, animals were placed individually in standard cages for 15 min for three days, after these three adaptation periods, the formalin test was carried out. 50 μL of 5% formalin solution was injected subcutaneously into the dorsal surface of the right hind paw. Pain behavior was monitored for a period of 60 min; the number of flinches/shakes of the injected paw was summed at 5-min intervals starting at time 0. Two phases of spontaneous flinches behavior were observed: phase 1 began immediately after formalin injection to 10 min thereafter and phase 2 began at time 10 min. A maximum response was observed around 20–45 min after the formalin injection.

#### Experimental protocol

First, three adaption sessions were carried out for each animal before testing. After this, paracetamol or saline was intraperitoneally administered, and 15 min later, the antagonist (WAY 100,635 or SB 216,641) or agonist (8-OH-DPAT or CP 93,129), of 5-HT_1A_ or 5-HT_1B_ receptors respectively, or saline was subcutaneously injected. Formalin was administered 30 min after paracetamol administration and immediately the animal was placed in individual behavioural cage, the test was recorded for 60 min.

### Statistical analysis

Results were expressed as mean ± SEM of the number of flinches/shakes of the phase 1 and 2 of the formalin test. The data obtained from the formalin test were statistically analyzed using two-way ANOVA. The factors of variation were paracetamol treatment and serotonin antagonist or agonist treatment. Subsequent one-way ANOVA was performed followed by Student-Newman-Keuls′ test, a value of *p* < 0.05 was considered to be significant.

## Results

### Antinociceptive effect of paracetamol in formalin test

**Fig. 1:**
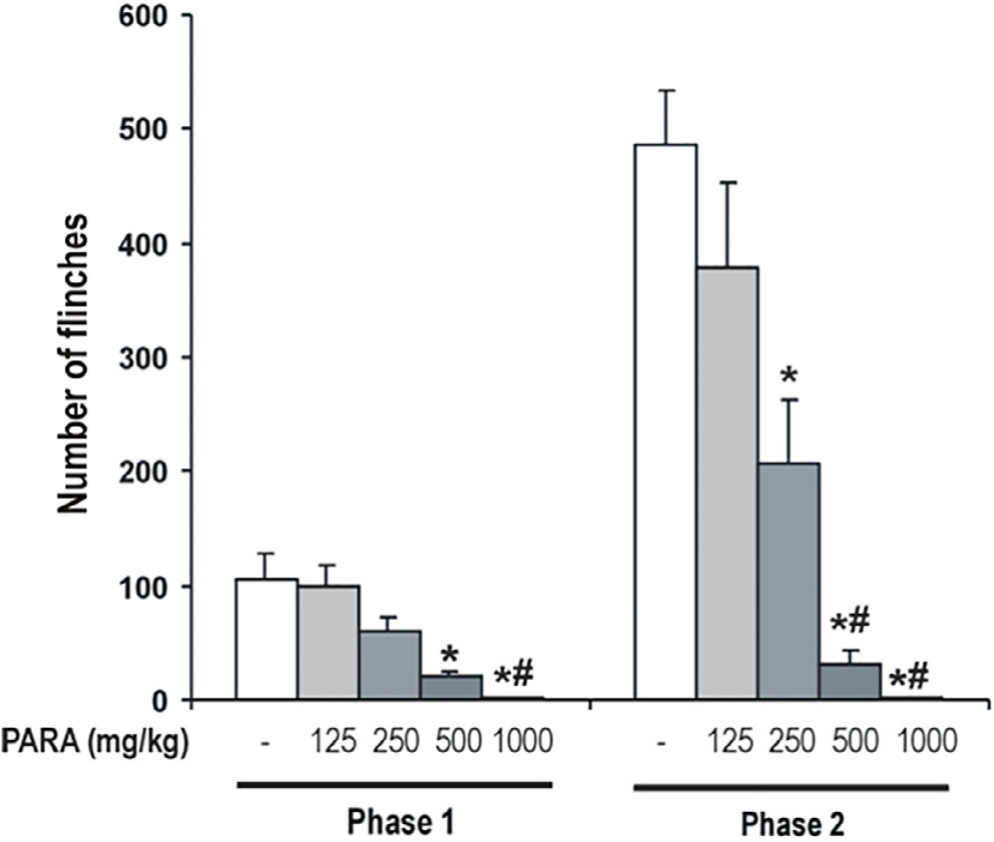
Antinociceptive effect of paracetamol in formalin test. Different doses of paracetamol (125, 250, 500 and 1000 mg/kg) or saline were administered 30 min before of formalin test. Two phases of spontaneous flinches/shakes behavior were observed over the 60 min test period. Error bars represent the SEM of 7–8 animals/group. * p < 0.05 vs saline, # p < 0.05 vs PARA 125 and 250 mg/kg as assessed by one-way ANOVA followed by a Newman-Keuls post-test.

The antinociceptive effect of paracetamol was evaluated in the formalin test in rats. One-way ANOVA showed a significant effect of treatment in both phases of the formalin test (Phase 1: F_4,34_ = 10.88, *P* < 0.001; phase 2: F_4,34_ = 19.21, *P* < 0.001). Paracetamol induced an increase in pain response latency in a dose-related manner in both phases of the formalin test (Fig. 1). In phase 1, paracetamol 250 mg/kg, but not 125 mg/kg, induced a non significant decrease of the number of flinches. Whereas, paracetamol 500 mg/kg and 1000 mg/kg induced a significant decreased of the number of flinches when compare to saline treated group. Also, dose of 1000 mg/kg induced significant decreased of the number of flinches compared to the doses of 125 and 250 mg/kg of paracetamol. In phase 2, paracetamol 125 mg/kg induced a non significant decreased of the number of flinches. However, paracetamol 250, 500 and 1000 mg/kg induced a significant decreased of the number of flinches compared to saline treated group, also, the decrease of number of flinches induced by paracetamol 500 and 1000 mg/kg was significant compared to paracetamol 125 and 250 mg/kg.

Therefore, paracetamol exert an antinociceptive effect in a dose-dependent manner in the formalin test.

We chose the doses of 125 and 250 mg/kg of paracetamol, with weak analgesic effect, to examine its association with specific antagonist of the serotonin receptors subtypes, 5-HT_1A_ and 5-HT_1B_. The doses of 500 and 1000 mg/kg of paracetamol, with strong analgesic effect, were chosen to test its combination with specific agonists of the 5-HT_1A_ and 5-HT_1B_ receptors.

### Involvement of 5-HT_1A_ receptors in the antinociceptive effect of paracetamol

#### Effect of 5-HT_1A_ antagonist on antinociceptive effect of paracetamol

The effect of WAY 100,635 0.8 mg/kg (selective 5-HT_1A_ antagonist) on the antinociceptive effect of paracetamol 125 mg/kg (a non effective analgesic dose) and 250 mg/kg (a weak antinociceptive dose) was evaluated using the formalin test. A Two-way ANOVA revealed a significant effect of paracetamol (Phase 1: F_2,48_ = 6.43, *P* < 0.004; Phase 2: F_2,48_ = 25.37, *P* < 0.0001) and WAY 100,635 (Phase 1: F_1,48_ = 10.10, *P* < 0.003; Phase 2: F_1,48_ = 13.05, *P* < 0.001). No significant effect was observed in the interaction of both treatments (Phase 1: F_2,48_ = 0.83, N.S.; Phase 2: F_2,48_ = 0.37, N.S.).

**Fig. 2:**
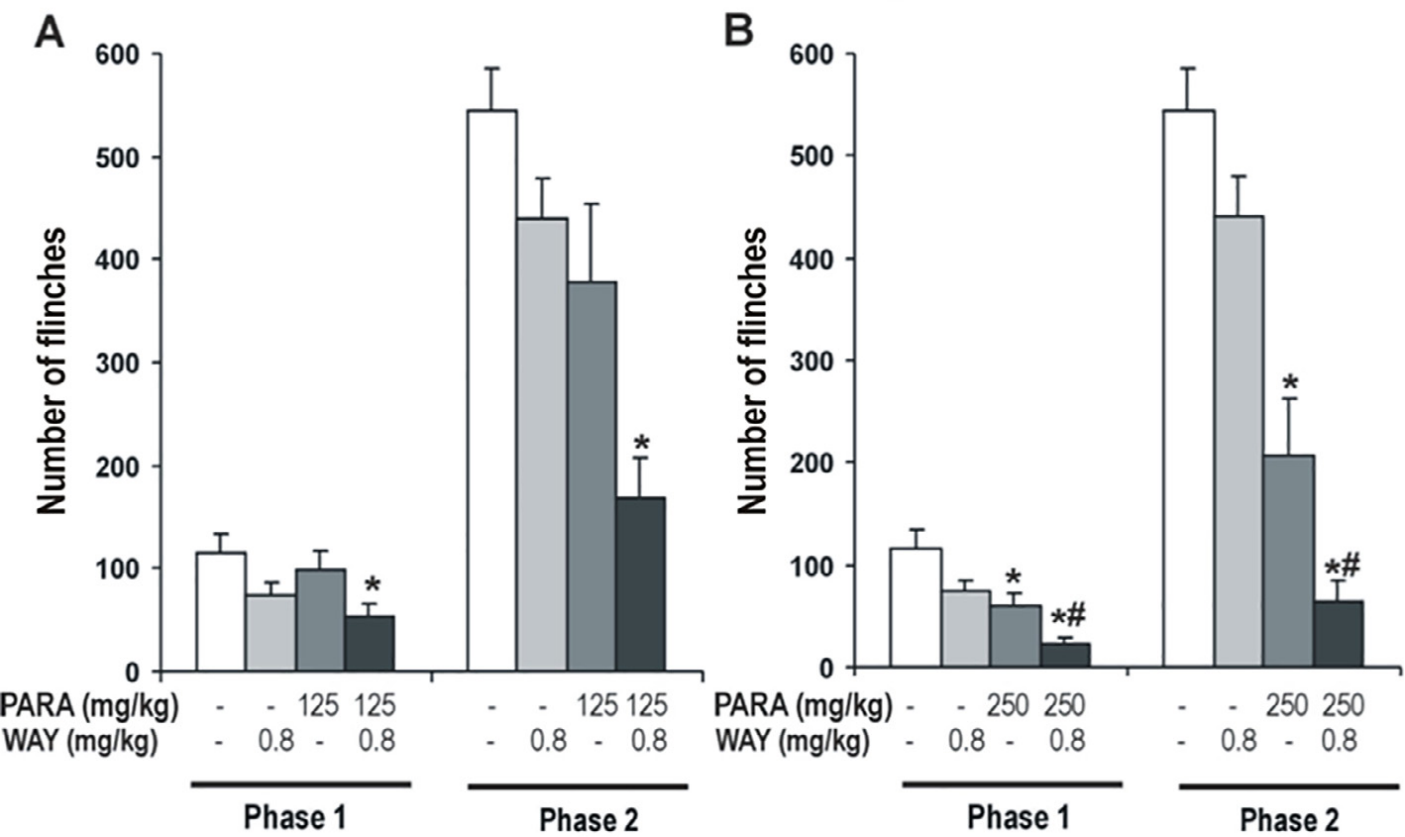
Effect of WAY 100,635 (0.8 mg/kg), 5-HT_1A_ antagonist, on antinociceptive effect of paracetamol (125 and 250 mg/kg; *A and B respectively*). WAY100635 was administered 15 min after paracetamol administration. Formalin test was performed 30 min after of paracetamol administration. Two phases of spontaneous flinches/shakes behavior were observed over the 60 min test period. Error bars represent the SEM of 8–9 animals/group. * p < 0.05 vs saline, # p < 0.05 vs PARA 250 mg/kg as assessed by one-way ANOVA followed by a Newman-Keuls post-test.

When the analgesic effect of a non effective analgesic dose of paracetamol (125 mg/kg) was measured, one-way ANOVA showed a significant effect of the treatment of 125 mg/kg of paracetamol in phase 2 (F_3,30_ = 9.57, *P* < 0.0001), although the treatment was non significant in phase 1 (F_3,30_ = 2.46, N.S.) of the formalin test. Likewise, the number of flinches in paracetamol-WAY 100,635 treated animals was modified (Fig. 2A). This decreased of the number of flinches induced by WAY 100,635 in rats receiving paracetamol 125 mg/kg was statistically significant compared to saline treated animals in both phases. WAY 100,635 had no effect in saline-treated animals.

Similarly, the analgesic effect of paracetamol was measured with a weak antinociceptive dose. In one-way ANOVA of 250 mg/kg of paracetamol revealed a significant effect of the treatment in both phases of the formalin test (Phase 1: F_3,30_ = 8.39, *P* < 0.0001; Phase 2: F_3,30_ = 27.34; *P* < 0.0001) (Fig. 2B). Also it could be observed as WAY 100,635 increased the antinociceptive effect of paracetamol, it induced a significant decrease of the number of flinches in both phases respect to paracetamol 250 mg/kg treated animals.

These results show that 5-HT_1A_ antagonist, WAY 100,635, increase the antinociceptive effect of paracetamol and, consequently, 5-HT_1A_ receptors maybe involved in the antinociceptive mechanism of paracetamol.

#### Effect of 5-HT_1A_ agonist on antinociceptive effect of paracetamol

The effect of 8-OH-DPAT 0.125 mg/kg (a selective 5-HT_1A_ agonist) on the antinociceptive effect of paracetamol 500 and 1000 mg/kg was evaluated. Two-way ANOVA showed a significant effect of paracetamol (Phase 1: F_2,48_ = 16.75, *P* < 0.0001; Phase 2: F_2,48_ = 113.75, *P* < 0.0001) and the interaction of both treatment (Phase 1: F_2,48_ = 4.26, *P* < 0.021; Phase 2: F_2,48_ = 3.57, *P* < 0.037) in phases 1 and 2 of the formalin test. But no significant effect of 8-OH-DPAT was observed in both phases of the formalin test (Phase 1: F_1,48_=, N.S.; Phase 2: F_1,48_ = 3.47, N.S.).

**Fig. 3:**
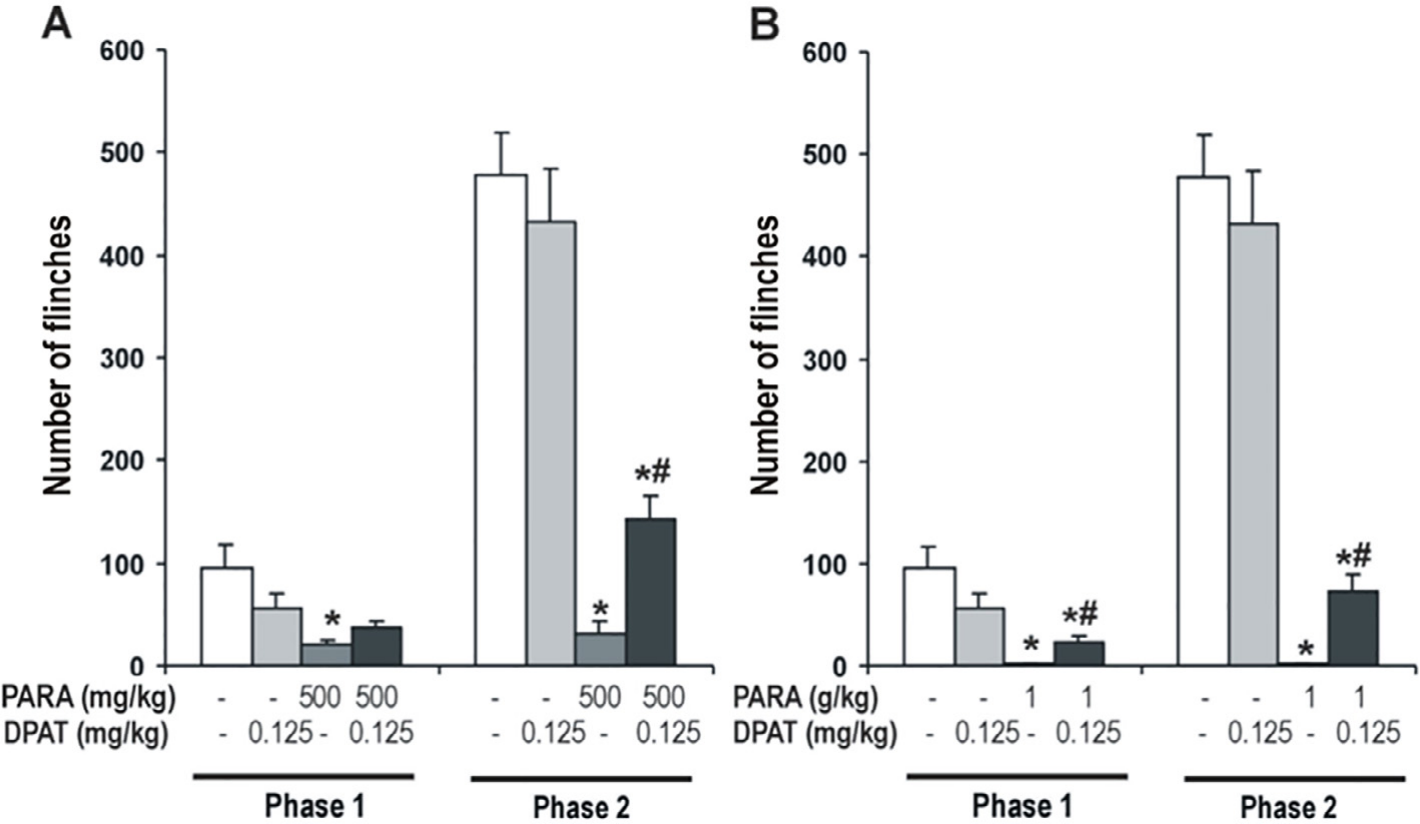
Effect of 8-OH-DPAT (0.125 mg/kg), 5-HT_1A_ agonist, on antinociceptive effect of paracetamol (500 mg/kg and 1 g/kg; *A and B respectively*). 8-OH-DPAT was administered 15 min after paracetamol administration. Formalin test was performed 30 min after of paracetamol administration. Two phases of spontaneous flinches/shakes behavior were observed over the 60 min test period. Error bars represent the SEM of 8–9 animals/group. * p < 0.05 vs saline and DPAT, # p < 0.05 vs PARA 500 mg/kg and 1 g/kg as assessed by one-way ANOVA followed by a Newman-Keuls post-test.

One-way ANOVA showed a significant effect of 8-OH-DPAT on the antinociceptive effect of 500 mg/kg of paracetamol in both phases (Phase 1: F_3,31_ = 5.43, *P* < 0.005.; Phase 2: F_3,31_ = 36.17, P < 0.0001) of the formalin test (Fig. 3A). In phase 1, 8-OH-DPAT induced a non significant increase of the number of flinches in paracetamol-treated animals, however produced a significant increase in animals receiving paracetamol in the second phase. 8-OH-DPAT had no effect in saline treated animals (Fig. 3A).

Similarly, experiments were repeated with 1000 mg/kg of paracetamol and one-way ANOVA revealed a significant effect of 8-OH-DPAT on the antinociceptive effect of paracetamol in both phases of the formalin test (Phase 1: F_3,31_ = 9.41, *P* < 0.0001; Phase 2: F_3,31_ = 49.53; *P* < 0.0001) (Fig. 3B). 8-OH-DPAT modified the antinociceptive effect of paracetamol, it induced a significant increase of the number of flinches in both phases in paracetamol 1000 mg/kg treated animals.

These results show that 5-HT_1A_ agonist, 8-OH-DPAT, decreased the antinociceptive effect of paracetamol suggesting the possible role of the 5-HT_1A_ receptors in the antinociceptive mechanism of paracetamol.

### Involvement of 5-HT_1B_ receptors in the antinociceptive effect of paracetamol

#### Effect of 5-HT_1B_ antagonist on antinociceptive effect of paracetamol

The effect of SB 216,641 0.8 mg/kg (a selective 5-HT_1B_ antagonist) on the antinociceptive effect of paracetamol 125 mg/kg (a non effective dose) and 250 mg/kg (a weak antinociceptive dose) was tested. Two-way ANOVA revealed a significant effect of paracetamol (Phase 1: F_2,56_ = 9.09, *P* < 0.0001; Phase 2: F_2,56_ = 16.54, *P* < 0.0001) and no significant effect of SB 216,641 (Phase 1: F_1,56_ = 2.42, N.S.; Phase 2: F_1,56_ = 2.33, N.S.) was observed, nor any interactions between both treatments (Phase 1: F_2,48_ = 0.83, N.S.; Phase 2: F_2,48_ = 0.37, N.S.).

**Fig. 4:**
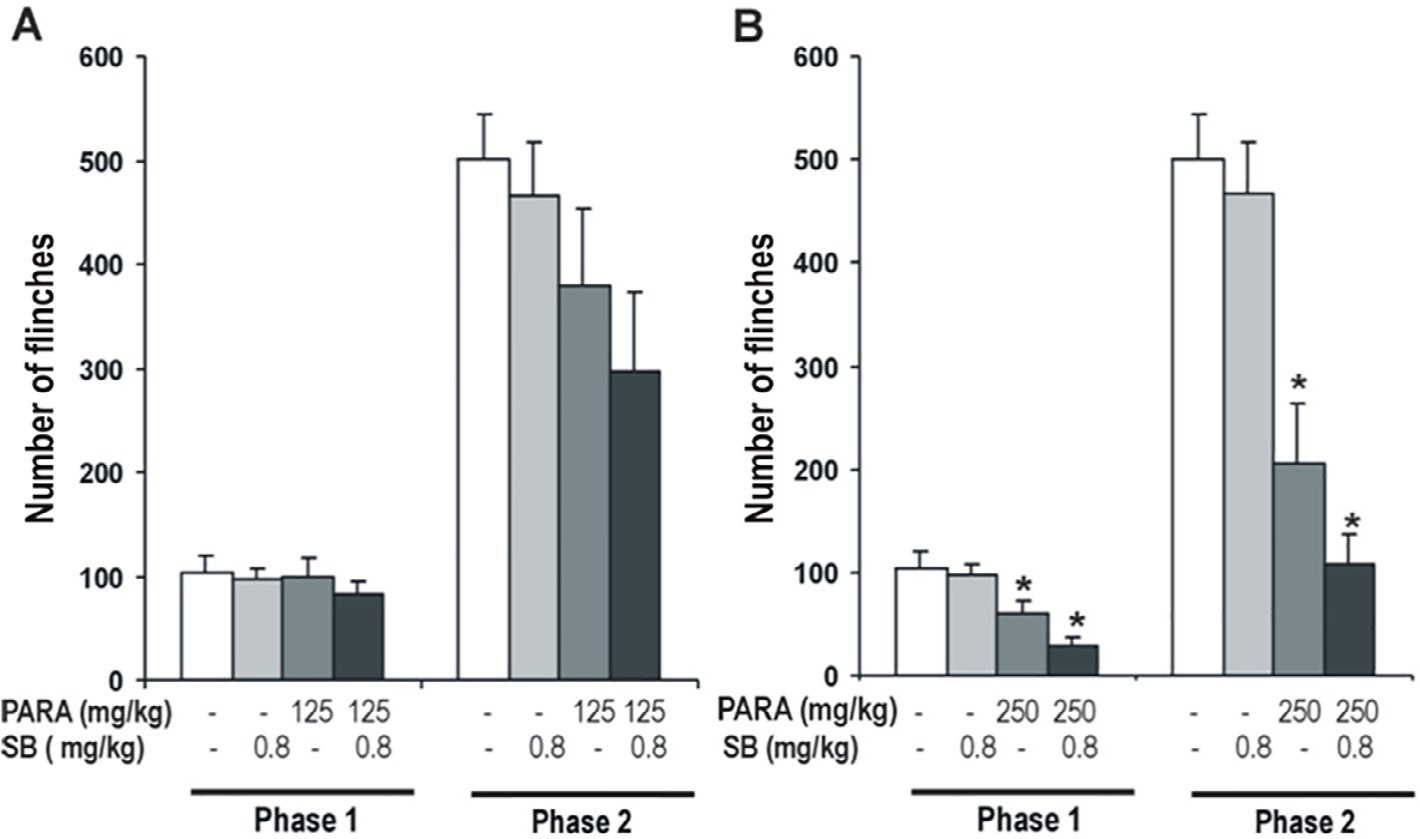
Effect of SB 216,641 (0.8 mg/kg), 5-HT_1B_ antagonist, on antinociceptive effect of paracetamol (125 and 250 mg/kg; *A and B respectively*). SB 216,641 was administered 15 min after paracetamol administration. Formalin test was performed 30 min after of paracetamol administration. Two phases of spontaneous flinches/shakes behavior were observed over the 60 min test period. Error bars represent the SEM of 10–11 animals/group. * p < 0.05 vs saline and SB as assessed by one-way ANOVA followed by a Newman-Keuls post-test.

When the analgesic effect of 125 mg/kg of paracetamol with a selective 5-HT_1B_ antagonist SB 216,641 was measured, one-way ANOVA showed a non-significant effect of SB 216,641 on the antinociceptive effect of paracetamol in both phases (Phase 1: F_3,37_ = 0.41, N.S.; Phase 2: F_3,37_ = 2.25, N.S.) of the formalin test. SB 216,641 induced a non-significant increased of the antinociceptive effect in paracetamol treated animals (Fig. 4A). SB 216,641 had no effect in saline treated animals.

In the same manner experiments were repeated with 250 mg/kg of paracetamol and one-way ANOVA revealed a significant effect of SB 216,641 on the antinociceptive effect of paracetamol in both phases of the formalin test (Phase 1: F_3,37_ = 7.78, *P* < 0.0001; Phase 2: F_3,37_ = 18.79; *P* < 0.0001) (Fig. 4B). SB 216,641 modified the antinociceptive effect of paracetamol. It induced a non-significant decrease of the number of flinches in both phases in paracetamol 250 mg/kg treated animals.

According to results, SB 216,641 0.8 mg/kg, 5-HT_1B_ antagonist, not show a clear effect on the antinociceptive action of paracetamol.

#### Effect of 5-HT_1B_ agonist on antinociceptive effect of paracetamol

The effect of CP 93,129 0.125 mg/kg (a selective 5-HT_1B_ agonist) on the antinociceptive effect of paracetamol 500 y 1000 mg/kg was examined. Two-way ANOVA revealed a significant effect of paracetamol (Phase 1: F_2,54_ = 61.97, *P* < 0.0001; Phase 2: F_2,54_ = 196.49, *P* < 0.0001) and no significant effect of CP 93,129 (Phase 1: F_1,54_ = 0.34, N.S.; Phase 2: F_1,54_ = 1.31, N.S.) was observed, nor any interactions between both treatments (Phase 1: F_2,54_ = 0.16, N.S.; Phase 2: F_2,54_ = 2.70, N.S.).

**Fig. 5:**
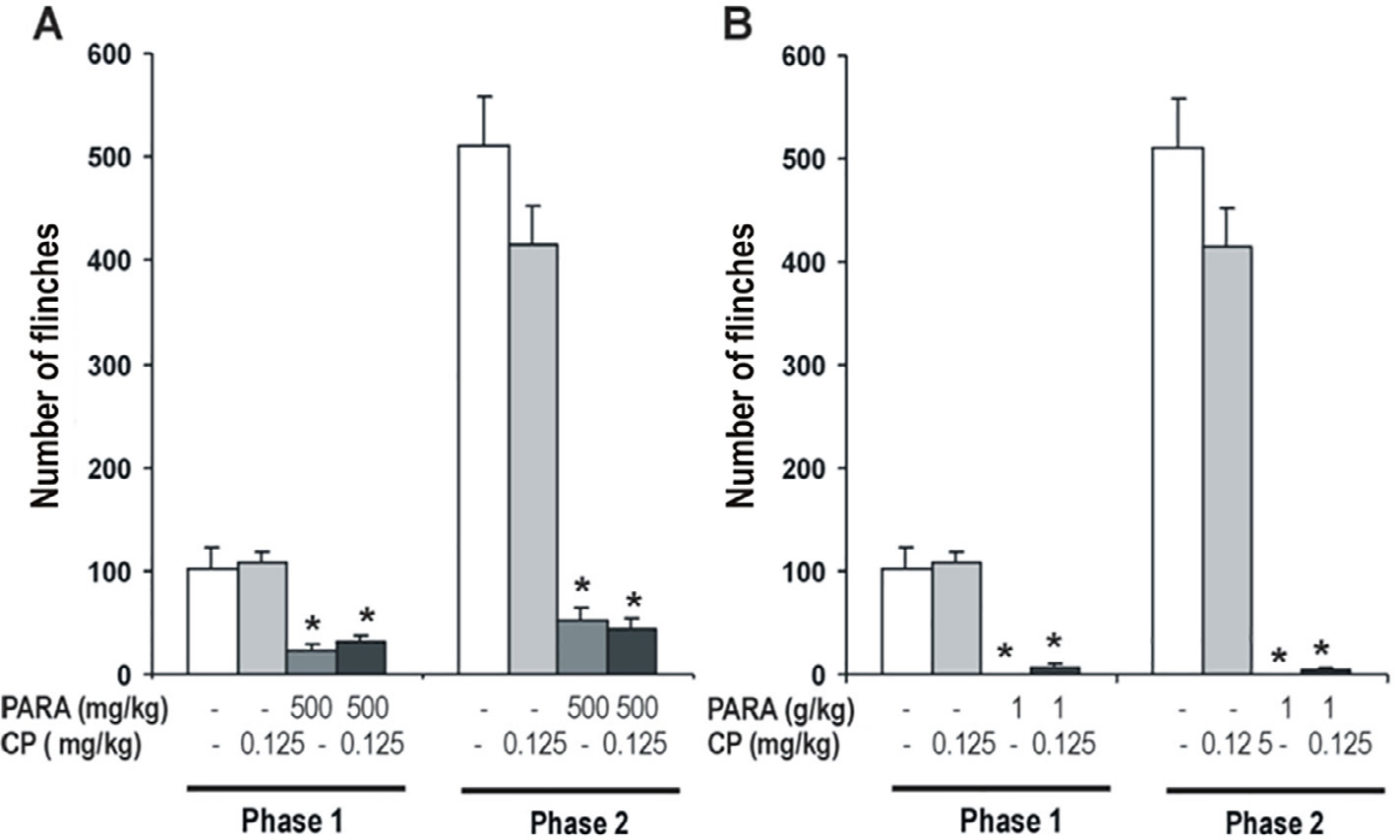
Effect of CP 93,129 (0.125 mg/kg), 5-HT_1B_ agonist, on antinociceptive effect of paracetamol (500 mg/kg and 1 g/kg; *A and B respectively*). CP 93,129 was administered 15 min after paracetamol administration. Formalin test was performed 30 min after of paracetamol administration. Two phases of spontaneous flinches/shakes behavior were observed over the 60 min test period. Error bars represent the SEM of 9–10 animals/group. * p < 0.05 vs saline and CP as assessed by one-way ANOVA followed by a Newman-Keuls post-test.

Antinociceptive effect of 500 mg/kg of paracetamol was studied and one-way ANOVA showed a significant effect in both phases (Phase 1: F_3,35_ = 17.57, *P* < 0.005.; Phase 2: F_3,35_ = 60.04, P < 0.0001) of the formalin test. CP 93,129 had no effect in saline treated animals and did not modify the antinociceptive effect produced by paracetamol in both phases of the formalin test (Fig. 5A).

Similar results were observed with 1000 mg/kg of paracetamol, one-way ANOVA revealed that this dose had a significant antinociceptive effect in both phases of the formalin test (Phase 1: F_3,35_ = 28.37, *P* < 0.0001; Phase 2: F_3,35_ = 76.74; *P* < 0.0001) (Fig. 5B). CP 93,129 modified weakly the antinociceptive effect of paracetamol, but it induced a non-significant increase of the number of flinches in both phases in paracetamol treated animals.

In line with the above results, CP 93,129 0.125 mg/kg, 5-HT_1B_ agonist, not show a significant effect on the antinociceptive action of paracetamol.

## Discussion

In this study, we evaluated whether the 5-HT_1A_ and 5-HT_1B_ receptors are involved in the antinociceptive effect of paracetamol in the rat model of formalin-induced pain. Flinches were used to quantify formalin-induced behaviours since they provide a reliable correlation of pain in the awake, freely moving rat. The behavioural response to the injection of formalin is biphasic, with an acute phase followed by tonic phase. It has been suggested that the early phase is caused by a direct effect of formalin on nociceptors, whereas the second phase is due to an inflammatory process (Le Bars et al., 2001). Therefore, the antinociceptive activity of paracetamol can be evaluated immediately after formalin injection. Our results showed a weak antinociceptive effect at doses of 125 and 250 mg/kg of paracetamol, and a strong analgesic effect at doses of 500 and 1000 mg/kg of paracetamol in both phases. In line with previously published data, our results confirmed that paracetamol is able to induce a dose-dependent antinociceptive activity in the formalin test in rat (Dogrul et al., 2012, Gong et al., 2011).

To study whether the 5-HT_1A_ and 5-HT_1B_ receptors are involved in the antinociceptive effect of paracetamol, we evaluated if the blockade of the 5-HT_1A_ or 5-HT_1B_ autoreceptors by different antagonists (WAY 100,635 and SB 216,641, respectively) can potentiate the antinociceptive effect of paracetamol and, in contrast, the activation of the 5-HT_1A_ or 5-HT_1B_ by different agonists (8-OH-DPAT and CP 93,129, respectively) reduced the antinociceptive effect induced by paracetamol. Our data clearly show that WAY 100,635, selective 5-HT_1A_ antagonist, potentiates the antinociceptive effect of paracetamol in the formalin test. While 8-OH-DPAT, selective 5-HT_1A_ agonist, reduced its analgesic effect in the same test. However, our results not show a clear effect on the antinociceptive effect of paracetamol when is administered SB 216,641, selective 5-HT_1B_ antagonist, or CP 93,129, selective 5-HT_1B_ agonist.

The 5-HT_1A_ receptors have a somatodendritic location on 5-HT neurons of the midbrain raphe nuclei (autoreceptors) and on neurons postsynaptic to 5-HT nerve terminals, mainly in cortico-limbic areas that exerts a pronounced inhibitory influence upon the release of 5-HT throughout the CNS. Also, 5-HT_1A_ can be localised at the spinal cord, a diversity of analgesiometric paradigms has been employed and numerous behavioural studies have reported hyperalgesia upon spinal administration (Alhaider and Wilcox, 1993, Bardin et al., 2000). Stimulation of 5-HT_1A_ receptors also attenuates induction of antinociception by the antidepressant, clomipramine (Ardid et al., 2001). While some authors (Bardin et al., 2003, Colpaert et al., 2002) have demonstrated that a 5-HT_1A_ agonist, F13640, induced central analgesia in different analgesimetric test. Our results showed that the 5-HT_1A_ antagonist, WAY 100,635, induced an increase of antinociceptive effect of paracetamol in the formalin test at a low dose. These results are supported by different studies showing that alprenolol and WAY 100,635 induced antinociception in the writhing test mice (Millan, 1994, Millan et al., 1996). Also, other study show paracetamol or venlafaxine with WAY 100,635 led to a significant antinociceptive effect (Bonnefont et al., 2005). A recent study, show the role of 5-HT_1A_ in the antinociceptive effect of paracetamol, but suggest that spinal 5-HT_7_ receptors are involved in a central antinociceptive and antihyperalgesic effect of paracetamol (Dogrul et al., 2012). However, these results are conflicting, because some studies have shown that 5-HT_1A_ agonists induced antinociception. For instance, the antinociceptive effect of several 5-HT_1A_ agonist, as 8-OH-DPAT, has been demonstrated in the formalin test (Granados-Soto et al., 2010) as well as the antinociceptive effect of buspirone increased the licking latency in the hot-plate test in mice (Liang et al., 2003). These data seem to indicate that the results obtained in the experiments depends on the nature of the noxious stimuli and, consequently, to the nature of the afferent fibre involved and the administration route of the drug, thus, the serotonergic system pharmacologic is very complex in controlling nociceptive pathways.

Regarding the 5-HT_1B_ receptors, they act as terminal receptors and are involved in the presynaptic regulation of the release of 5-HT. But at spinal level these receptors are principally situated post-synaptically (Sari, 2004). The ability of autoreceptors to regulate extracellular levels of 5-HT during release has made them the focus of much interest. Our results show that SB 216,641, selective 5-HT_1B_ antagonist, modified weakly the antinociceptive effect of paracetamol and CP 93,129, selective 5-HT_1B_ agonist, not produce a clear effect in the antinociceptive effect of paracetamol. In our study, CP 93,129 was used to a doses of 0.125 mg/kg, however, there are studies that show that CP 93,129 at 0.250 mg/kg or 2 mg/kg doses s.c., decreased the antinociceptive effect of paracetamol in the hot plate-test (Roca-Vinardell et al., 2003, Sandrini et al., 2003). Therefore, higher doses of CP 93,129 could have an effect on the antinociceptive effect of paracetamol in the formalin test.

Moreover, many data indicate that locomotion may influence nociception, but the changes have not always been well elucidated (Le Bars et al., 2001). In this line, it would be interesting to evaluate the spontaneous motor activity after treatment with the drugs used both alone or in combination.

As previously mentioned, it is well-known that the descending serotonergic pathway origins at supraspinal sources. The predominant proportion of serotonergic neurons arises from the nucleus raphe magnus, although, a modest sources of serotonergic neurons from dorsal raphe nucleus innervates the spinal cord as well (Wang and Nakai, 1994). Traditionally, actions of 5-HT in the descending serotonergic pathway have been considered to suppressed the nociceptive transmission (Basbaum and Fields, 1978). Nevertheless, opposite actions (pronociceptive or antinociceptive) of 5-HT have been described depending on the 5-HT receptor and localisation of the specific 5-HT receptor types. Thus, activation of the 5-HT_1A_, 5-HT_1B_, 5-HT_1D_ and 5-HT_7_ receptors tends to be antinociceptive, whereas the 5-HT_2A_ and 5-HT_3_ receptor tend to promote nociception (Rahman et al., 2009, Suzuki et al., 2004). Antinociception in mice produced by rostroventromedial medulla morphine was blocked by spinal 5-HT_7_ antagonist and hyperalgesia produced by rostroventromedial medulla cholecystokinin was blocked by a spinal 5-HT_3_ antagonist (Dogrul et al., 2009). In other studies in mice, systemic 5-HT_7_ agonists blocked hyperalgesia, whereas 5-HT_7_ antagonists elicited enhanced pain (Brenchat et al., 2009). In this regard, antinociceptive or pronociceptive mechanism can be generated as result of the activation of the descending serotonergic pathway indicating an important serotonergic role for bidirectional pain modulation.

### Conclusion

In conclusion, the results of this study suggest that the antinociceptive effect of paracetamol in the formalin test can be enhanced mainly by antagonist of the 5-HT_1A_ receptors, and, perhaps, by antagonist compounds of the 5-HT_1B_ receptors. Consequently, these receptors have a role in the analgesic effect of paracetamol. Thus, this study provides a possible and a promising pharmacological combination for the development of a new analgesic strategy.

### Fundings

This work was supported by grants from the “Ministerio de Economía y Competitividad” (MINECO), co-financed by “Fondo Europeo de Desarrollo Regional” FEDER “A way to build Europe” (SAF2015-68647-R); the “Centro de Investigación Biomédica en Red de Salud Mental-CIBERSAM” (Spain; G18); the “Consejería de Economía, Innovación, Ciencia y Empleo de la Junta de Andalucía” (Spain; CTS-510 and CTS-7748); “Fundación Progreso y Salud de la Junta de Andalucía” (PI-0080-2017); and Fundación Española del Dolor (PI2015-FED-007).

### Conflict of interest

There is no conflict of interest for any of the authors.
